# Complete Genome Sequence of Klebsiella quasipneumoniae subsp. *similipneumoniae* Strain IF3SW-P1, Isolated from the International Space Station

**DOI:** 10.1128/mra.00476-22

**Published:** 2022-06-23

**Authors:** Natasha S. Sushenko, Nitin K. Singh, Daniel L. Vellone, Scott W. Tighe, Brian P. Hedlund, Kasthuri Venkateswaran, Duane P. Moser

**Affiliations:** a Department of Hydrologic Sciences, Desert Research Institute, Las Vegas, Nevada, USA; b School of Life Sciences, University of Nevada Las Vegas, Las Vegas, Nevada, USA; c Jet Propulsion Laboratory, California Institute of Technology, Pasadena, California, USA; d Vermont Integrative Genomics Resource, University of Vermont, Burlington, Vermont, USA; e Nevada Institute of Personalized Medicine, University of Nevada Las Vegas, Las Vegas, Nevada, USA; University of Rochester School of Medicine and Dentistry

## Abstract

The 5.2-Mb circular genome of Klebsiella quasipneumoniae subsp. *similipneumoniae* strain IF3SW-P1, isolated from the International Space Station, was sequenced using Oxford Nanopore Technologies. The genome lacks a megaplasmid typical of hypervirulent and multidrug-resistant Klebsiella strains but does contain a chromosomally encoded OqxAB efflux pump associated with carbapenem resistance.

## ANNOUNCEMENT

In 2014, two phylogroups of the opportunistic pathogen Klebsiella pneumoniae were described as the novel species Klebsiella quasipneumoniae (1). Since its definition as a species, *K. quasipneumoniae* has emerged as an understudied human pathogen with hypervirulent, multidrug-resistant (MDR), carbapenem-resistant, and hypermucoviscous strains isolated from both hospital-borne and community-acquired infections (2–5). Considering its prevalence on the International Space Station (ISS) (6), the newly recognized pathogenicity of *K. quasipneumoniae* increases concerns about the consequences of this species being exposed to the stresses of spaceflight, which are known to trigger bacterial virulence and antimicrobial resistance (7–10).

Strain IF3SW-P1 was isolated from the surface of the foot panel of the Advanced Resistive Exercise Device (ARED) on the ISS on 4 March 2015 (11) using a standard spread plate method on Reasoner’s 2A (R2A) agar and archived in glycerol cryostocks (6). For this study, strain IF3SW-P1 was subcultured from cryostock and grown to late exponential phase in Trypticase soy broth (TSB) at 37°C; genomic DNA was then extracted using the DOE Joint Genome Institute bacterial genomic DNA isolation protocol (12).

Oxford Nanopore Technologies sequencing was performed using a GridION MK1 sequencer on a R10.4 flow cell (FLO-MIN112) with a library synthesized from Q20+ EA (early access) ligation reagents (SQK-LSK112-XL). The raw reads were base called using MinKNOW v29.10.8, with a mean quality score of 16.3 and a mode of 18.03. The genome was assembled, circularized, and polished using Flye v2.9 with the parameters *–nano-hq* and *–read-error 0.03* for the Q20+ data (13). The Flye-generated assembly contains two contigs, one 5.2-Mb circular chromosome and one 3-kb linear fragment confirmed via BLASTN v2.12.0 to be 99.8% identical to Escherichia coli strain Q4552 plasmid pECQ4552_IHU08 (GenBank accession number CP077071.1) (14). Notably, the genome does not encode any virulence- or drug resistance-associated plasmids, such as *bla*KPC and Inc(FII), which are known to occur in Klebsiella species (15).

The genome was identified as *K. quasipneumoniae* subsp. *similipneumoniae* by calculating the average nucleotide identity (ANI) using the EzBioCloud calculator compared to the two subspecies’ type strains, *K. quasipneumoniae* subsp. *quasipneumoniae* 01A030^T^ (ANI, 96.63%) and *K. quasipneumoniae* subsp. *similipneumoniae* 07A044^T^ (ANI, 99.03%) (16). Strain IF3SW-P1 is also related to but distinct from eight previously published draft genomes of *K. quasipneumoniae* strains isolated from the ISS, with >99% ANI for all (6).

The assembly was annotated using RASTtk v1.3.0 (17) as part of the Pathosystems Resource Integration Center (PATRIC) v3.6.12 (18). Predicted virulence genes on the chromosome include *iutA*, which encodes a ferric aerobactin receptor, although the gene encoding the associated siderophore aerobactin (*iucA*) is not present (19). The IF3SW-P1 genome also contains genes for the multidrug resistance efflux pump OqxAB, associated with carbapenem resistance in K. pneumoniae (20, 21). OqxAB is reported to be associated with resistance to benzalkonium chloride, a quaternary ammonium compound used as a disinfectant on the ISS (11). Default parameters were used for all software unless otherwise specified. Additional assembly and annotation information is listed in Table 1.

**TABLE 1:** Assembly and annotation information

Characteristic	Data
Strain name	IF3SW-P1
ISS sampling date	4 March 2015
Location	ARED foot panel
Nearest species	*K. quasipneumoniae* subsp. *similipneumoniae* 07A044^T^
ANI (%)	99.03
No. of raw reads	364,351,976
Genome size (bp)	5,238,176
*N*_50_ (bp)	5,238,176
No. of contigs	2 (1 chromosomal, 1 linear fragment)
Median coverage (×)	62
G+C content (%)	58.06
No. of coding sequences	4,998
GenBank accession no.	CP092121
SRA accession no.	SRR17974437
GeneLab accession no.	GLDS-470

### Data availability.

The genomic assembly and raw reads have been deposited at GenBank (accession number CP092121) and the Sequence Read Archive (SRR17974437). These data are also available at NASA GeneLab (GLDS-470).
